# First case of human gongylonemosis in France

**DOI:** 10.1051/parasite/2013007

**Published:** 2013-02-14

**Authors:** Bernard Pesson, Christel Hersant, Jean-François Biehler, Ahmed Abou-Bacar, Julie Brunet, Alexander W. Pfaff, Hubert Ferté, Ermanno Candolfi

**Affiliations:** 1 Laboratoire de Parasitologie et de Mycologie Médicale, Plateau Technique de Microbiologie, Hôpitaux Universitaires de Strasbourg 1 rue Koeberlé 67000 Strasbourg France; 2 Laboratoire d’Analyses Médicales Biehler 5 Grand rue 68170 Rixheim France; 3 Institut de Parasitologie et de Pathologie Tropicale de la Faculté de Médecine, EA-7292 DHPI, Université de Strasbourg 3 rue Koeberlé 67000 Strasbourg France; 4 Faculté de Pharmacie de Strasbourg 74 route du Rhin 67401 Illkirch France; 5 USC Anses « Vecpar » EA 4688, UFR de Pharmacie, Université de Reims Champagne-Ardenne 51 rue Cognacq-Jay 51096 Reims France

**Keywords:** *Gongylonema*, human infection, zoonosis, France, case report

## Abstract

*Gongylonema* spp. are cosmopolitan spirurid nematodes that are common parasites of wild and domesticated mammals and birds. *Gongylonema pulchrum* Molin, 1857 is most common in ruminants, where it invades mucosa and submucosa of the mouth, tongue, oesophagus and forestomachs. It extremely rarely occurs in man, and fewer than 60 cases have been reported worldwide. We report a case from the Alsace region, which appears to be the first case of human gongylonemosis described in France.

## Abstract (fr)

Les nématodes du genre *Gongylonema* sont des spiruridés cosmopolites parasites fréquents dans de nombreux mammifères et oiseaux sauvages ou domestiques. *Gongylonema pulchrum* Molin, 1857 est l’espèce la plus souvent rapportée chez les ruminants, dans la muqueuse et la sous-muqueuse de leur bouche, langue, œsophage et rumen. Il n’est que très exceptionnellement retrouvé chez l’homme. Moins de 60 cas ont été décrits à travers le monde. Nous rapportons dans cet article le premier cas français, découvert en Alsace.

## Case report

During the summer of 2012, a healthy 48-year-old man felt the presence of a moving, worm-like organism in his mouth. Initially, the patient would occasionally feel, but not see, this mass at different sites: cheek, palate, gums and internal surface of the lower lip. The sensation would subside after several hours without leaving any visible lesions and without being accompanied by any associated localized or generalized symptoms.

The patient had no medical history. He is a resident of Alsace, France, and had not travelled abroad. He works as a maintenance service agent in a harbour on the river Rhine. He reported not to have changed his lifestyle, especially not his diet, in the recent past. He also had no knowledge of having accidentally ingested an intermediate insect host. He consulted a doctor and all results of the clinical examination fell within the normal range. Haematology investigation revealed no abnormalities, particularly no elevated eosinophil count, and no microfilariae were seen using stained blood films; the filariasis serology was negative. No medical treatment was initiated.

**Figure 1.**
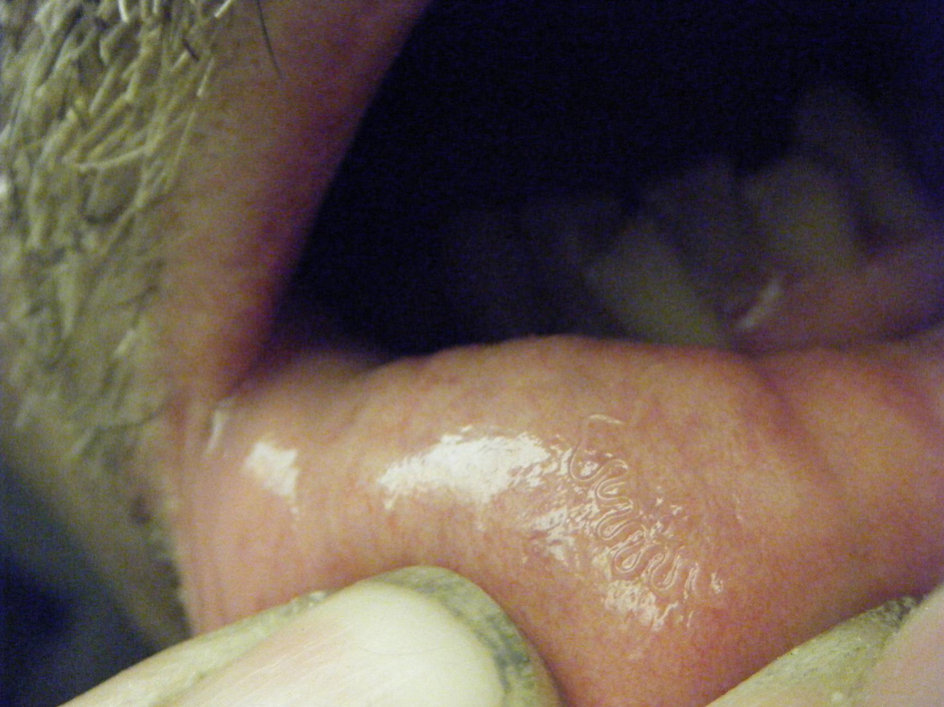
Serpentine path of *Gongylonema pulchrum* in lip mucosa.

After 3 weeks of migration, the thread-like worm installed itself on the inner surface of the lower lip (Figure 1), allowing the patient to extract it by tongue pressure firstly, then using his fingers. He placed the parasite in alcohol and submitted it to a medical laboratory. The biologist in charge sent the specimen to the Laboratory of Parasitology and Medical Mycology of the Strasbourg University Hospital for identification. No recurrence, lesions, bleeding or other symptoms have since been experienced by the patient.

## Specimen

**Figure 2.**
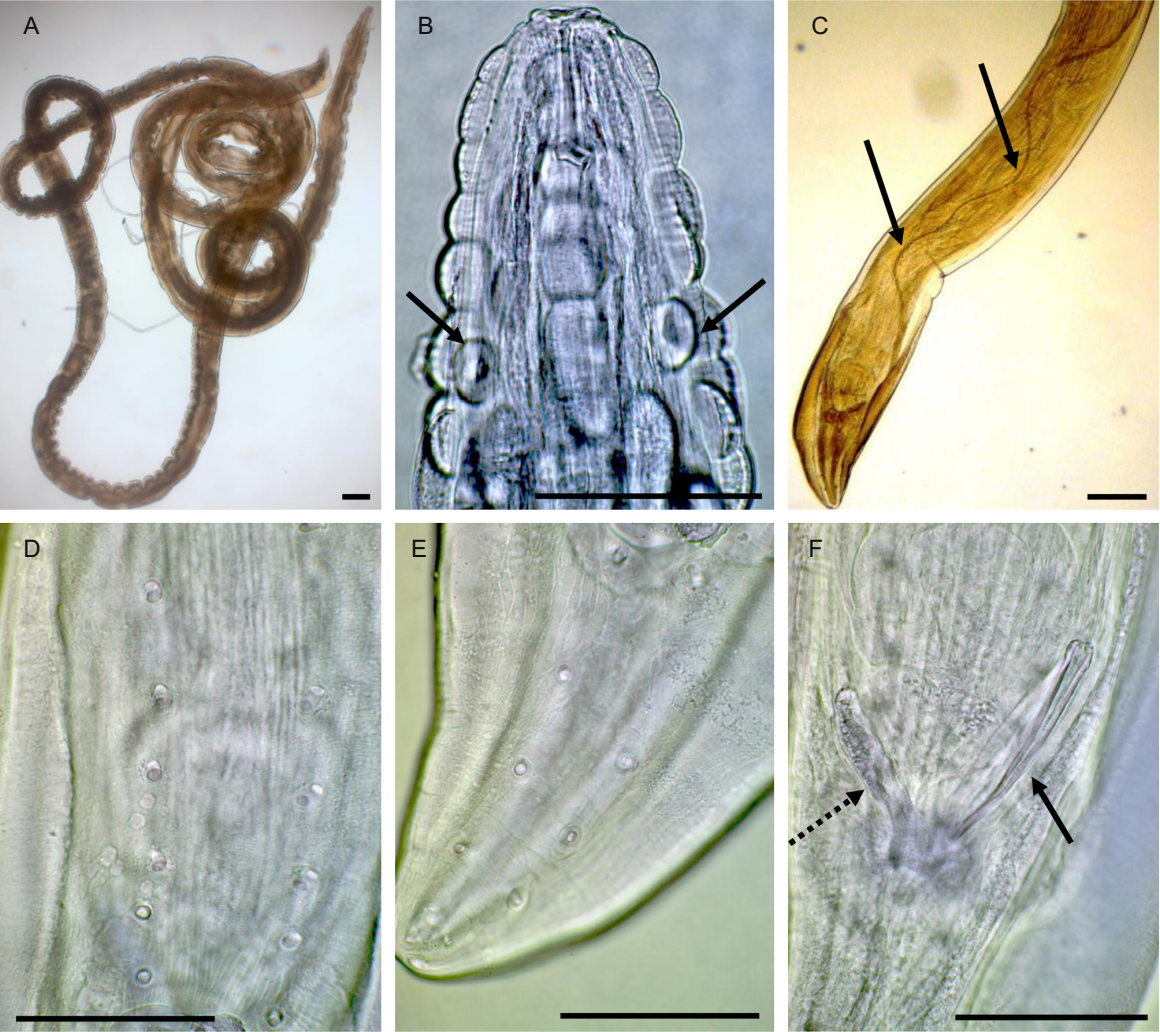
*Gongylonema pulchrum*. A, entire male. B, cuticular bosses and deirids (arrows). C, posterior end and left spicule (arrows). D, precloacal papillae. E, postcloacal papillae. F, posterior end: right spicule (arrow) and gubernaculum (dotted arrow). Bars: 100 μm.

The entire worm has been removed and stored in 70% ethanol; it was cleared in lactophenol and examined under a compound microscope. The morphological characteristics of this single male correspond to those of *Gongylonema pulchrum* Molin, 1857 (measurements in micrometres unless otherwise stated): Body length 39 mm, maximum width 250 (Figure 2A). Anterior end with longitudinal rows of verruciform cuticular bosses (Figure 2B), extending 950 posteriorly from apex, deirids at 125 from anterior end (Figure 2B). Posterior end with asymmetrical caudal alae (Figure 2C), left ala 750 long, right ala 625 long; six precloacal (Figure 2D) and five postcloacal (Figure 2E) pairs of caudal papillae; spicules very unequal; length of right spicule, left spicule and gubernaculum 120, 7.5 mm and 85, respectively (Figure 2C and F).

## Discussion

*Gongylonema* spp. are heteroxenous parasites of the upper digestive tract of many species of birds and mammals. They are most often described in ruminants, but also in rodents, bears or monkeys [10]. The adult worms occur in the stomach and in the oesophagus where they burrow and migrate in the mucosa, forming a characteristic sinuous pathway. The females lay embryonated eggs which pass in the faeces where they are swallowed by coprophagous insects, mainly dung beetles and cockroaches; more than 70 species are possible intermediate hosts [11]. First-stage larvae hatch and moult twice before developing into the infective third-stage, first in the haemocoel, but finally most infective larvae become encapsulated in the muscles. This development in the intermediate host takes about 30 days [1]. The definitive host becomes infected by ingestion of the insect or parts of it containing third-stage larvae. These larvae may emerge from intermediate hosts that fall into water or are crushed on vegetables and remain infectious for a month [2, 6]. The existence of paratenic vertebrate hosts, such as small mammals or poultry, has been discussed by Euzeby [5]. Definitive hosts could become infected when ingesting their viscera.

The migration of the worm in its definitive host is not well studied. Alicata [1] suggested that larvae excyst in the stomach and invade its wall, then migrate anteriorly to the oesophagus or oral cavity where they reach sexual maturity in about 2 months. Due to the way of transmission, human gongylonemosis is rare. As in all previously reported cases, it was impossible to determine how the infection was acquired in the present case: the patient had no history of insect contact or recent travel abroad. The presumed route of transmission is an accidental ingestion of food or water contaminated by infected insects. It is noticeable that the role of paratenic hosts suggested by Euzeby [5] is never taken into consideration in medical literature.

Haruki *et al.* [6] analysed 48 cases reported from all continents, since the first mention of human infection in 1850, and 41 of these were attributed to *G. pulchrum*. Subsequently, Urch *et al.* [12] reported a new case from Germany and in 2006, Molavi *et al.* [9] recorded the first case from Iran. In 2012, the most recent case was reported from the United States by Ayala and Yencha [3]. Most of these cases involve young women affected by a single immature *G. pulchrum* female, located in the oral cavity. In contrast, our case describes a middle-aged man affected by a single *G. pulchrum* male. To our knowledge, this is the first case reported in France.

*Gongylonema pulchrum* is frequent in ruminants and pigs where it is considered as relatively harmless, usually causing only chronic oesophagitis. It is susceptible to the main anthelmintic agents used in the veterinary control of nematode infections, particularly levamisole [8]. In man, the worms are most often found in different areas of the oral cavity, such as the lips or gingiva, where they cause transient inflammatory migrating or moving lumps in the buccal mucosa. Frequently no lesions or any other symptoms are noticed. Exceptionally, pruritus, salivation, toothache, pharyngitis and oesophagitis are described. The chronicity and recurrence of stomatitis could cause nervous disturbances [2, 6, 14]. Haematological values remain within the normal range, particularly with no hypereosinophilia being observed.

Treatment consists of manually removing the worms using the fingers or a needle or forceps: one end of the mature worm often extends into the oral cavity [2]. Anti-inflammatory or local antiseptic applications might facilitate the migration of the worms from the mucosa [7, 13]. The infection is often caused by one or two worms and the symptoms disappear within a few days after removal of the worm without any further therapy. Some patients have been treated with albendazole [4, 13]. Different studies have investigated the benefit of using this anthelmintic: it does not favour the elimination of the parasite, but can eradicate other possibly present nematodes [15]. In the present case, the patient extracted the worm himself and did not receive any anthelmintic treatment.

*Gongylonema pulchrum* is a common parasite of animals which rarely infects humans. Despite its worldwide distribution, it is an unfamiliar parasite, little-known by clinicians and medical biologists. The typical symptoms described by the patients, such as the sensation of a migrating thread-like form and the localization in the oral cavity, must prompt a careful clinical examination of the mouth. Any worm-like objects should be removed and studied under a microscope, where the presence of the typical verruciform cuticular bosses would easily confirm the final diagnosis of gongylonemosis.
